# IgE sensitization to house dust mite and cockroach allergens in asthmatic and allergic patients in the tropics

**DOI:** 10.3389/falgy.2025.1727880

**Published:** 2025-12-17

**Authors:** Randy Reina, Nathalie Acevedo, Miguel Ángel Caballero, Isabel Gil, Ramon Lopez-Salgueiro, Luis Caraballo

**Affiliations:** 1 Institute for Immunological Research, University of Cartagena, Cartagena, Colombia; 2 Clinica Respiratoria y de Alergias, Cartagena, Colombia; 3 Laboratorio de Diagnóstico Biológico del Servicio de Alergia del Hospital Universitari i Politècnic La Fe, Valencia, Spain; 4 Departamento de Ciencias Biomedicas, Universidad Cardenal Herrera CEU, Valencia, Spain

**Keywords:** cockroach, house dust mite, IgE sensitization, cross-reactivity, shellfish allergy, shrimp, component resolved diagnosis

## Abstract

**Introduction:**

House dust mite (HDM) allergens are major triggers of IgE-mediated asthma in tropical regions, yet the role of cockroach allergens and their cross-reactivity with HDM remains unclear. Cross-reactivity among invertebrate allergens is a common challenge in daily practice, especially to define primary sensitizers and reactions of clinical relevance. Multiplexed arrays in molecular allergology constitute a useful tool for better detection and interpretation of cross-reactions.

**Methods:**

We assessed specific IgE levels and skin prick test reactivity to the American cockroach and HDM allergens in cohorts of allergic and asthmatic patients from Cartagena, Colombia, using ImmunoCAP™, skin testing, and multiplex molecular allergology (ALEX2).

**Results:**

Cockroach sensitization was present in 29%–40% of patients but elicited significantly lower IgE responses and smaller skin test wheals compared with HDM. Most cockroach-sensitized individuals were cosensitized to HDM, with limited recognition of cockroach molecular components. Mean specific IgE levels to cockroach were 2.1 kU/L ranging from 0.1 to 25.8 kU/L. The majority of patients had IgE levels in Class 1 (0.35–0.70 kU/L) or Class 2 (0.70–3.5 kU/L). In the ALEX2 array, most cockroach-sensitized patients (by skin tests) did not recognize the *Periplaneta americana* extract (Per a) or other cockroach allergens in the array, and instead they recognized HDM allergens and the extracts of crustaceans and mollusks. Only one patient recognized the Per a extract, cockroach tropomyosin (Per a 7), and tropomyosins in HDM (Blo t 10, Der p 10), shrimp (Pen m 1), and Anisakis simplex (Ani s 3) together with other allergens in crustaceans and mollusks. Interestingly, IgE reactivity to cross-reactive allergens like arginine kinase, myosin light chain, and sarcoplasmic calcium-binding protein was not detected. Cockroach sensitization was not associated with worsened asthma control or lung function but correlated with higher shrimp-specific IgE in patients reporting shellfish allergy.

**Discussion:**

HDM allergens induce stronger IgE responses than cockroach in this tropical population, indicating HDM as the primary sensitizer. Cockroach sensitization often reflects cross-reactivity and requires careful clinical evaluation to determine its relevance.

## 1 Introduction

House dust mite (HDM) allergy is confirmed with the history of typical IgE-mediated respiratory symptoms such as sneezing, nasal itching, congestion, rhinorrhea, coughing, and wheezing after exposure to house dust along with the evidence of IgE sensitization (a positive result in a skin prick test/provocation test and/or positive-specific IgE antibodies in serum) (1). In daily clinical practice, it is common to observe specific cutaneous IgE reactivity to HDM as detected by skin testing, but also to other invertebrates such as mosquitoes, ants, insects, cockroaches, and crustaceans (2, 3). Although the clinical relevance of HDM reactivity has been widely demonstrated (4), the importance of other invertebrate cosensitizations (including cockroach) needs more study. For instance, approximately 20% of HDM allergic patients show shellfish allergy (5) that is often linked to cross-reacting tropomyosins found in both HDM and shellfish (6, 7). While not all HDM/shrimp cosensitized patients are clinically allergic (8), the presence of specific IgE antibodies to tropomyosin suggests that inhaled tropomyosins from HDM are the primary sensitizer, and in some patients, may pose a higher risk of developing an allergic reaction to shellfish if consumed (9–11).

The mechanisms why HDM induce intense IgE responses in patients from humid tropical environments are still unclear, but the perennial exposure to HDM during the whole year is an important factor, since this selects genetically predisposed subjects. Cross-reactive allergens such as tropomyosin may be relevant due to the sequence similarity in epitopes at the C-terminus in several invertebrates (12). There is also evidence that helminth infections occurring in tropical settings can increase the intensity of the IgE response to HDM allergens or other bystander allergens (13), and helminth nematodes have cross-reacting allergens with HDM (14) and cockroaches (15). Also, it has been suggested that exposure and sensitization in the first decade of life to *Dermatophagoides* spp*.* induce “molecular spreading” to other allergenic invertebrates such as cockroach (16).

We previously found that specific IgE to *Periplaneta americana* is detectable in 20.8% of children at 6 years of age in Cartagena (Colombia), but a positive skin prick test (SPT) reactivity was observed in 4.9% of them (13). IgE reactivity to the cockroach allergen Bla g 2 was also detected in asthmatic patients of this population, although the levels were much lower to those measured to Der p 2 (17). Several studies support that sensitization to cockroach allergens is one of the strongest risk factors for the development of asthma in low-income urban populations worldwide (18–20) and is associated with a severe form of asthma, prone to steroid dependency, hospital admission, and emergency unit visits due to asthma exacerbations (21–23).

Approximately 4,000 species of cockroach have been described worldwide, but only a few are domiciliary cockroaches. The most studied species are *Blattella germanica* (German cockroach) and *P. americana* (American cockroach) (18). Even though the German cockroach has a worldwide distribution (24), it has been described that *B. germanica* predominate in temperate areas and *P. americana* predominate in tropical areas. This cockroach distribution can be explained by the fact that the German cockroach prefers cool and dry climates, whereas the American cockroach prefers hot and humid environments (18). Indeed, cockroaches are commonly found in the households in our region (25). IgE sensitization to the American cockroach usually occurs by inhalation, and to date, 20 allergens have been described, including Per a 1, Per a 2, Per a 5, Per a 7, Per a 9, and Per a 10 (26, 27). These allergens are found in cockroach skin flakes, secretions, eggshells, whole carcasses, and fecal matter, and can become airborne and cause allergies in susceptible individuals (28). Cockroach allergy is associated with IgE sensitization to multiple allergens that vary among individuals, with no single allergen being consistently immunodominant, and both IgE and T cell reactivity are highly variable, with unique subject-specific profiles (29), and allergen recognition has been found to be different across diverse populations (30). The rate of prevalence of IgE sensitization to cockroach allergens in Brazil ranges from 20% to 80% depending on the population studied (31–33), with about half of patients recognizing tropomyosin (Per a 7) as the most frequent allergen component in sensitized individuals (34).

There is growing interest about the binding of IgE to allergen component arrays in different geographical regions and its associations with clinical allergy. Studies indicate that HDM and cockroaches emerge as the two most prevalent sensitized aeroallergens among children diagnosed with asthma and/or allergic rhinitis in South Asian regions (35, 36). However, the frequency of cockroach sensitization in urban allergic patients from tropical areas and its relationship with other invertebrate allergens (including HDM and shrimp) are not complete. Therefore, the objective of this study is to analyze the frequency of cockroach-specific IgE sensitization in asthmatic and allergic patients from the tropical Caribbean city Cartagena de Indias (Colombia) and to compare cockroach IgE reactivity with HDM IgE reactivity.

## 2 Materials and methods

### 2.1 Study population

Cohort 1: The first was an observational, descriptive, and retrospective analysis of the results of IgE measurements to HDM and cockroach performed at the Respiratory and Allergy Clinic Laboratory between 24 June 2024 and 10 September 2025. Blood samples were obtained from patients attending consultations for allergic symptoms and who had a diagnosis or perceived diagnosis of asthma, rhinitis, atopic dermatitis, urticaria, and/or atopic rhinoconjunctivitis. At the time of blood sampling, there was no previous information about sensitizing agents. A blood sample was obtained by standard phlebotomy in dry tubes, and after clot formation, the samples were centrifuged and serum stored at −20°C until analysis. Patients signed an informed consent form for sample collection and the analysis of their demographic and clinical variables.

Cohort 2: This was an observational, descriptive, and cross-sectional analysis of 220 well-characterized asthmatic patients. These patients were recruited within an adult cohort on the “Studies of Pathogenesis of Asthma in the Tropics” (EPAT) study, conducted at the Institute for Immunological Research of the University of Cartagena. Patients were scheduled between August 2023 and November 2024 and underwent skin allergy testing with standardized extracts (see section 2.3 for details). Asthma was diagnosed according to the GINA guidelines and confirmed by a physician from the research staff. Lung function tests were performed by spirometry on all patients by trained healthcare personnel using an Easy-on-PC ultrasonic spirometer (NDD Medical Technologies, USA) in accordance with the guidelines established by the American Thoracic Society (ATS) (37). Exhaled fraction of nitric oxide (FeNO) was measured according to ATS guidelines by using NoBreath® (Bedfont® Scientific Ltd., UK) and levels reported in parts per billion (ppb) (38). There was no selection of participants based on the possible sensitizing source of allergen. A blood sample was taken from each participant in EDTA tubes, centrifuged at 1,000 *g* for 15 min at 4°C, and plasma stored at −80°C until use. This study was approved by the Ethics Committee of the University of Cartagena (Act 128, date 14/11/2019). All research activities were performed in accordance with the principles stated in the Declaration of Helsinki, and written informed consent to participate in this study and to publish details on study findings was obtained from each participant.

### 2.2 Specific IgE levels

In Cohort 1, specific IgE levels to HDM (*Dermatophagoides pteronyssinus* (d1), * Dermatophagoides farinae* (d2), * Blomia tropicalis* (d201), and *Periplaneta americana* (i206) were measured by using the ImmunoCAP™ system (Thermo Fisher Scientific/Phadia, Uppsala, Sweden) on the Phadia™ 200 instrument, employing commercially available CAP reagents. Positive IgE sensitization was defined by IgE levels ≥ 0.10 kU/L, a cutoff currently defined that can reliably detect IgE sensitization (39). Response (RU) values were extrapolated to a curve with six calibrators (0.001, 0.35, 0.70, 3.50, 17.5, and 100). Specific IgE levels were categorized into seven ImmunoCAP™ classes: Class 0 (<0.35 kU/L), Class 1 (0.35 to <0.70 kU/L), Class 2 (0.70 to <3.50 kU/L), Class 3 (3.50 to <17.5 kU/L), Class 4 (17.50 to <50 kU/L), Class 5 (50.00 to <100.00 kU/L), and Class 6 (>100.00 kU/L).

In Cohort 2, plasma samples from a subgroup of 20 asthmatic patients were analyzed using the Allergy Explorer 2 (ALEX2) platform (MacroArray Diagnostics, Vienna, Austria), according to the manufacturer's instructions. This semiquantitative multiplex assay enables the simultaneous quantification of total IgE and sIgE against 117 allergen extracts and 178 molecular components. Participants included in the ALEX2 analysis had a positive SPT result for the extracts of house dust mites (*B. tropicalis*, *D. pteronyssinus*, and *D. farinae*) with or without having a positive SPT result for *P. americana* (American cockroach). After the procedure, images of each cartridge were acquired and analyzed using the Multi Array Xplorer 45 k system (MAX 45 k) (40). The results were analyzed using the RAPTOR SERVER software, and IgE levels were expressed in kU_A_/L. Allergic sensitization was defined as the detection of an sIgE level ≥ 0.3 kU_A_/L as previously described (41, 42). sIgE levels determined by ALEX2 were reported in kU_A_/L and further categorized into five classes: Class 0 (<0.3 kU_A_/L), Class 1 (0.3 to <1 kU_A_/L), Class 2 (1 to <5 kU_A_/L), Class 3 (5 to <15 kU_A_/L), and Class 4 (≥15 kU_A_/L). The range of total IgE levels was 1–2,500 kU/L. Shrimp-specific IgE levels were measured in a group of samples of Cohort 2 (*n* = 48) using the f24 mix (*Pandalus borealis*, *Penaeus monodon*, *Metapenaeopsis barbata*, and *Metapenaeus joyneri*) by ImmunoCAP™ 200 following the manufacturer's instructions (Thermo Fisher, Uppsala, Sweden).

### 2.3 Skin prick tests

Skin prick tests were performed with standardized extracts of house dust mites, *D. pteronyssinus* (M601, 100 HEP/mL), *D. farinae* (M602, 100 HEP/mL), *B. tropicalis* (M608, 150 µg/mL), and *P. americana* (I703, 1,000 μg/mL), dog epithelium (E802, Can f 1 20 µg/mL) and cat epithelium (E801, 50 HEP/mL), grass mix (MG01, 50 HEP/mL), and *Aspergillus fumigatus* extract (P902, 25 μg/mL), with histamine (K200, 10 mg/mL) as positive control and 50% glycerol as negative control (K100), all obtained from Inmunotek S.L. (Alcalá de Henares, Madrid, Spain). The size of the wheal and erythema was documented in millimeters (mm). A skin prick test result was considered positive if the wheal diameter was 3 mm greater than negative control.

### 2.4 Statistical analysis

Statistical analyses were performed using GraphPad Prism software v.8.0.1 (Boston, MA, USA). Continuous (numerical) variables were assessed for normality using the Shapiro–Wilk test or the Kolmogorov–Smirnov test depending on the number of observations. Variables following a normal distribution were described using the mean and standard deviation (SD). Variables following a non-normal distribution were described using median and lower and upper quartiles (25th–75th). Frequencies were calculated using descriptive statistics. Specific IgE (sIgE) levels and papule diameters were expressed as continuous variables. The Wilcoxon signed rank test was used to compare sIgE levels and wheal diameters with HDM and American cockroach extracts within the same patient. Other continuous variables were compared using the Mann–Whitney *U*-test, as appropriate. Correlation between IgE levels was analyzed using Spearman’s correlation test. Differences in categorical variables were analyzed using Fisher’s exact test. A *P*-value below <0.05 was considered statistically significant.

## 3 Results

### 3.1 Comparative analysis on the strength of specific IgE levels to cockroach and HDM

**Figure 1:**
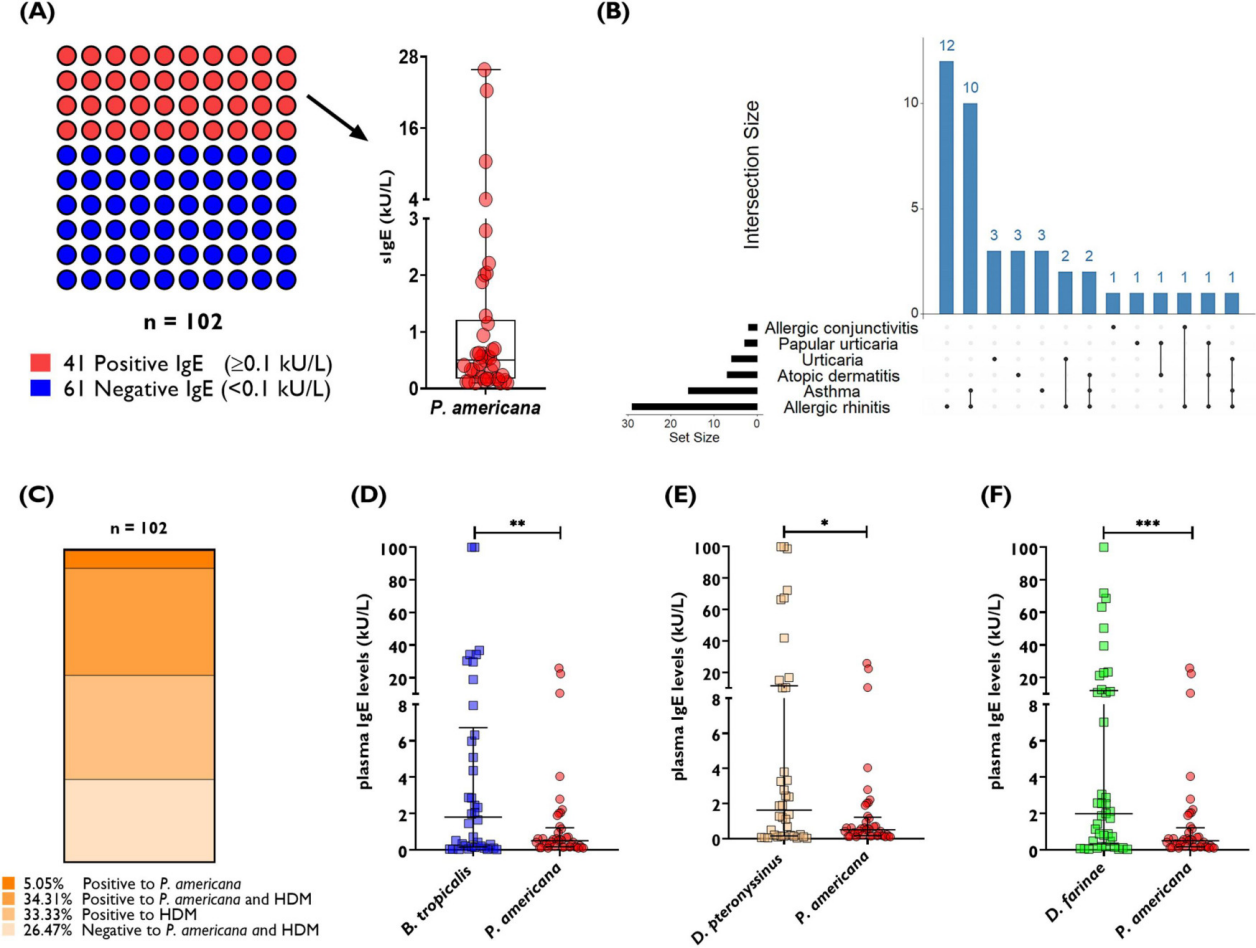
Comparative analysis of specific IgE levels to the American cockroach and HDM in a cohort of 102 allergic patients from Cartagena—Colombia (cohort 1). **(A)** Percentage of patients with a positive IgE result for *P. americana* and their plasma IgE levels in kU/L. **(B)** Distribution of allergic conditions in the study population. **(C)** Frequency of cosensitization to *P. americana* and HDM. Comparison of plasma IgE levels with **(D)** *B. tropicalis* and *P. americana*, **(E)** *D. pteronyssinus* and *P. americana*, and **(F)** *D. farinae* and *P. americana*. Each dot represents a patient. The error bars indicate the median and interquartile range. Wilcoxon matched-pairs signed rank test: *P = 0.01; **P = 0.001; ***P = 0.0005.

First, we analyzed the laboratory results of 102 allergic patients in whom a *P. americana* (i206) extract–specific IgE measurement was taken using ImmunoCAP™. We found that 41 patients tested positive to the American cockroach (≥0.1 kU/L), with a sensitization frequency of 40.2% (Figure 1A). If we used the cutoff of 0.35 kU/L, we found that the sensitization frequency to the American cockroach was 23.5% (*n* = 24/102). The mean age of the patients who tested positive to the cockroach was 20 years (range 2–68 years). With regard to the intensity of the IgE response, we found that cockroach-specific antibodies had a mean value of 2.1 kU/L with a range of 0.1–25.8 kU/L (Figure 1A). Allergic rhinitis (present in 29 of the 41 patients) and asthma (present in 16 of the 41 patients) were the two most common diagnoses in the cockroach-sensitized patients (Figure 1B). Five percent of the tested patients were sensitized to cockroach but not to HDM (Figure 1C).

**Figure 2:**
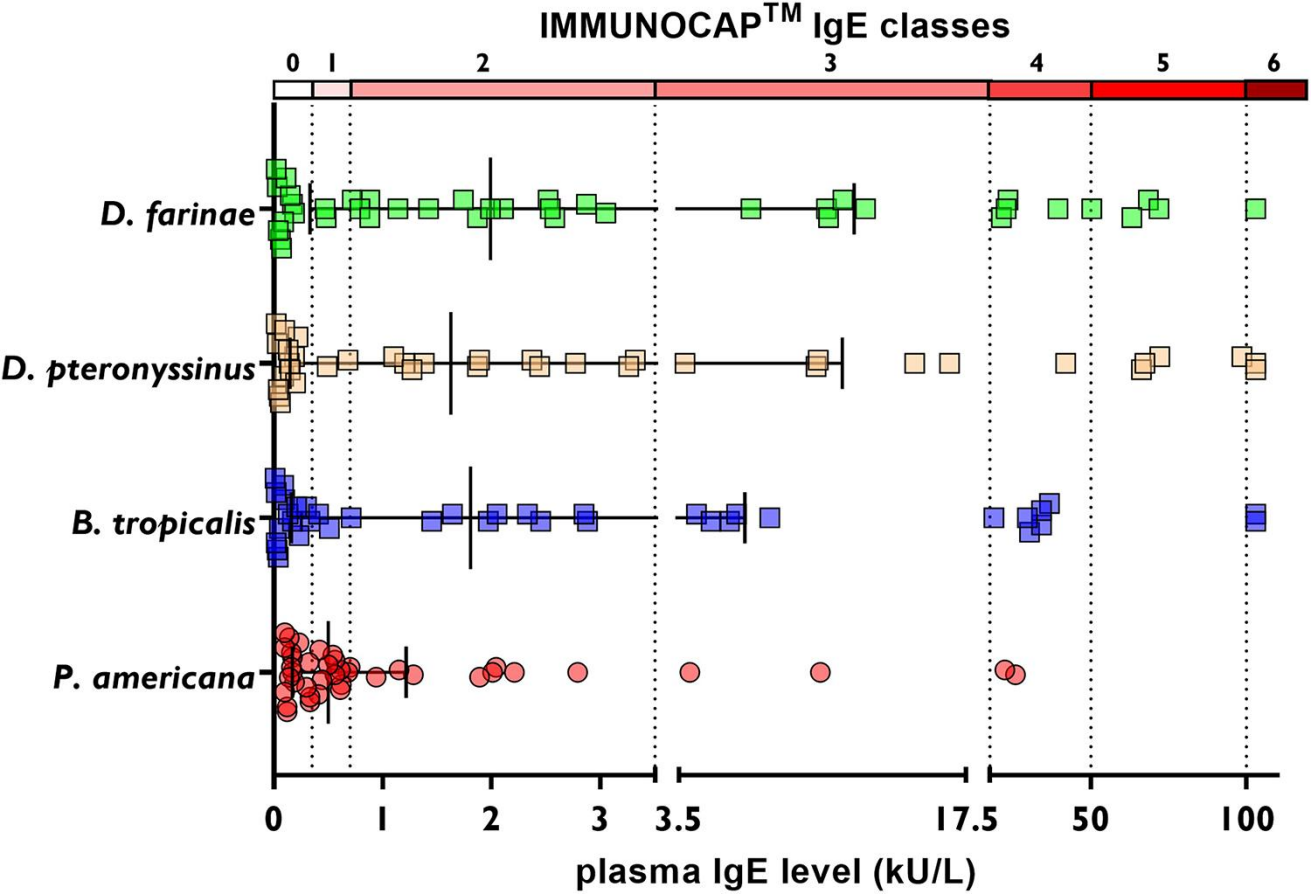
IgE classes according to plasma IgE levels determined by ImmunoCAP™ in cohort 1. The error bars indicate the median and IQR. Each dot represents an individual.

Comparing the intensity of the IgE response to the cockroach with the IgE response to the HDM extracts, we found that the levels of *B. tropicalis* (median IgE: 1.8 kU/L, IQR: 0.16–6.7 kU/L; *P* = 0.0011), *D. pteronyssinus* (median IgE: 1.6 kU/L, IQR: 0.15–11 kU/L; *P* = 0.012), and *D. farinae* (median IgE: 2.0 kU/L, IQR: 0.33–12 kU/L; *P* = 0.0005) were significantly higher compared with the response to the cockroach (Figures 1D–F). Moreover, the intensity of the response according to ImmunoCAP™ classes was higher to HDM compared with that to the cockroach, to which 17 patients were in the range of very low IgE levels (0.1–0.34 kU/L), 11 patients were in Class 1 (0.35 to <0.7 kU/L), 9 patients in Class 2 (0.7 to <3.5 kU/L), and only two patients in Class 3 (3.5 to <17.5 kU/L) and 2 in Class 4 (17.5 to <50 kU/L) (see Figure 2).

### 3.2 Frequency of skin IgE reactivity to cockroach extract in asthmatic patients

**Figure 3:**
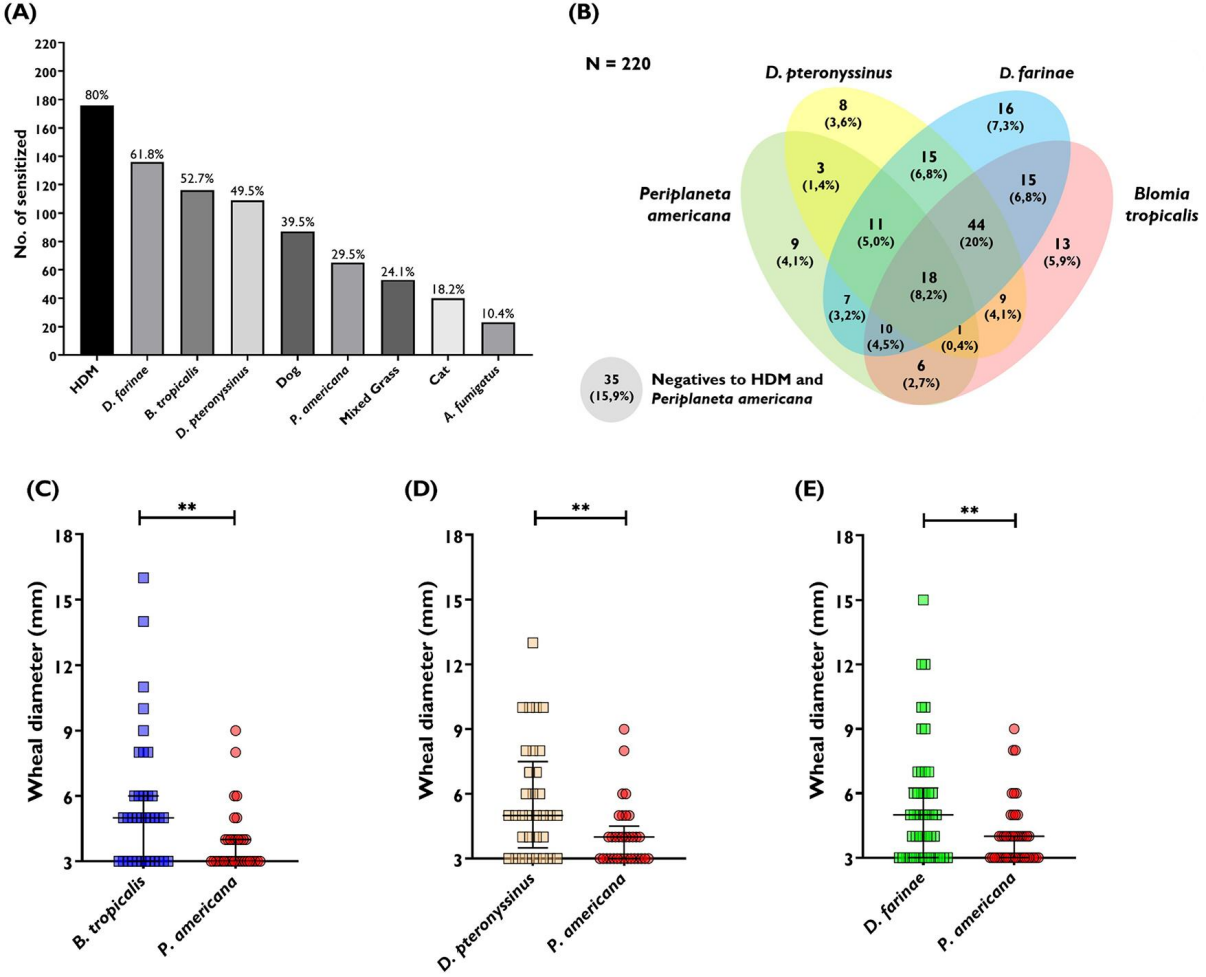
**(A)** Frequencies of IgE sensitization by SPT to a panel of common aeroallergens in a cohort of 220 asthmatic patients in a tropical region of Bolivar—Colombia (cohort 2). HDM indicates the percentage of patients with a positive skin prick test result for any of the three mites evaluated (*B. tropicalis*, *D. pteronyssinus*, or *D. farinae*). **(B)** Venn diagram on the number of positive patients for *P. americana*, *B. tropicalis*, *D. farinae,* and *D. pteronyssinus*;185 had a positive SPT result for HDM and/or the American cockroach out of the 220 tested, 35 patients had a negative SPT result for cockroach and HDM. **(C)** Wheal size of *P. americana* and *B. tropicalis* **(D)** wheal size of *P. americana* and *D. pteronyssinus* **(E)** wheal size of *P. americana* and *D. farinae*; patients with a positive SPT result for both cockroach and HDM were evaluated. **P < 0.01.

Between August 2023 and November 2024, a total of 220 adult patients with previous diagnosis of asthma were evaluated for allergen skin reactivity. The mean age of this cohort was 38 years (range 18–70 years); 176 patients (80%) were females, 160 patients (72.7%) had elevated FeNO with a median value of 42 ppb (IQR 24–78), and 84 patients (38.2%) had elevated blood eosinophil counts (≥300/μL) with a mean value of 303 eos/μL ± 216. Allergic rhinitis was the main comorbidity (reported in 89.5% of cases). The mean percentage predicted FEV_1_ value was 78% (±17) and the mean percentage predicted FVC value was 89% (±15). We found that 194 of these 220 patients (88.2%) had a positive SPT result for at least one of the aeroallergens evaluated, with sensitization to HDM allergens detected in 80% of patients. In addition, 87 patients (39.5%) were positive for dog epithelium; 65 patients (29.5%) were positive for *P. americana*; 53 (24.1%) were positive for a mixture of six grasses; 40 (18.2%) were positive for cat epithelium, and 23 (10.4%) were positive for *Aspergillus fumigatus*. The frequency of IgE reactivity is presented in Figure 3A.

### 3.3 Comparative analysis of skin IgE reactivity to cockroach and HDM extracts

**Figure 4:**
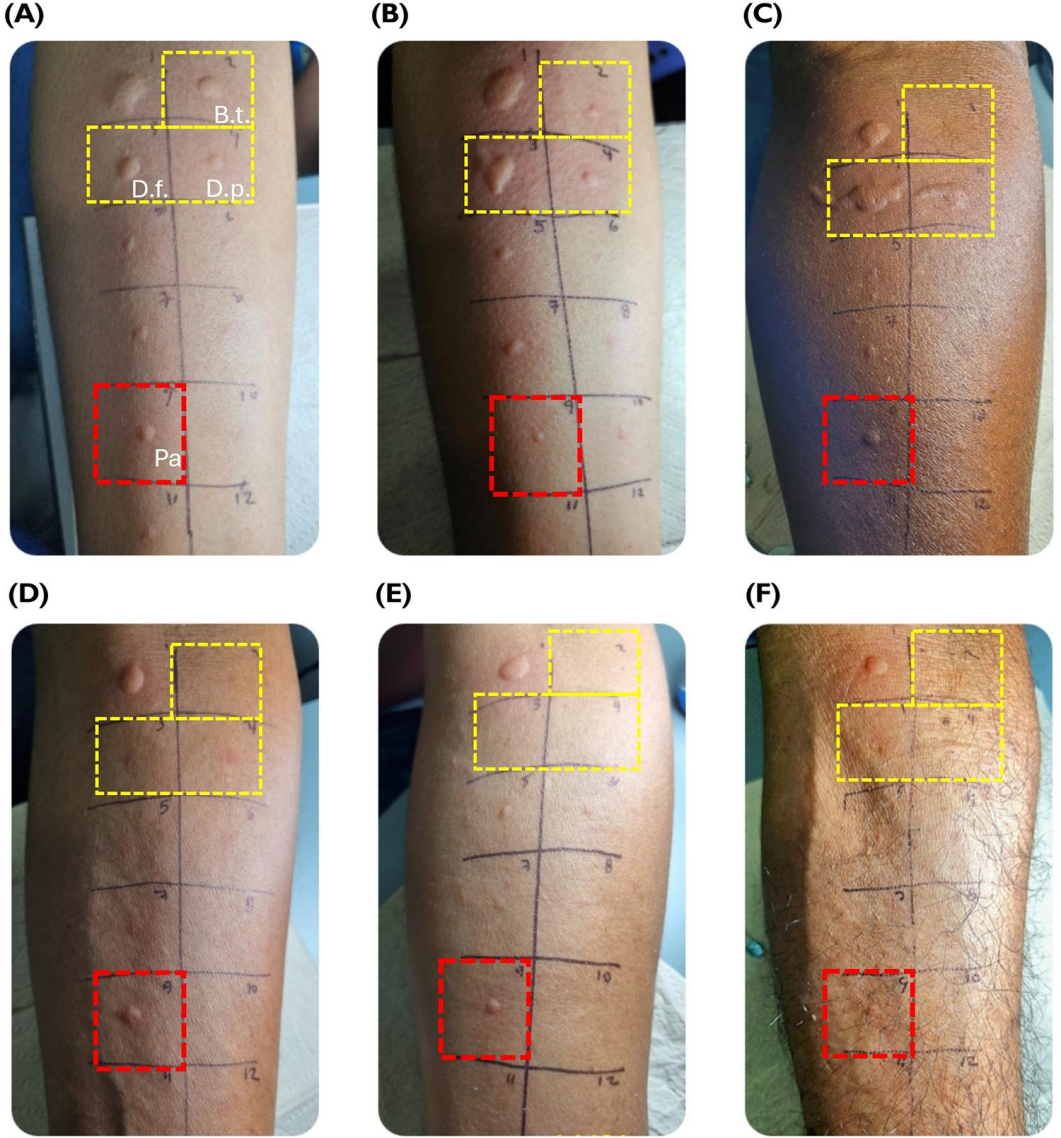
Skin reactivity to the American cockroach and HDM extracts in asthmatic patients from cohort 2. **(A–C)** show the SPT results of three patients positive for the American cockroach and HDM. **(D–F)** illustrate three patients positive for the cockroach but not for HDM. (1). Histamine, (2). *B. tropicalis*, (3). *D. farinae*, (4). *D. pteronyssinus*, (5). Dog epithelium, (6). Cat epithelium, (7). Mixed grass, (8). *A. fumigatus*, (9). *P. americana*, and (10). Negative control. The yellow boxes indicate HDM tests and the red box indicates the SPT result for *P. americana*.

The overlap of IgE sensitization between *P. americana*, *B. tropicalis*, *D. farinae*, and *D. pteronyssinus* is shown in Figure 3B. Of the 220 evaluated patients, 65 had a positive SPT result for the cockroach extract with a median wheal diameter of 3 mm (IQR 3–4 mm). Fifty-six patients with a positive SPT result for *P. americana* also had a positive SPT result for one or more of the HDMs evaluated. On the other hand, only nine individuals (13.8%) were sensitized to the cockroach extract and showed no cutaneous reactivity to the HDMs (Figure 3B). We found that wheal diameters induced by *B. tropicalis* (*n* = 35 positives, median diameter: 5 mm, IQR: 3–6 mm; *P* = 0.0024), *D. pteronyssinus* (*n* = 33 positives, median diameter: 5 mm, IQR: 3.5–7.5 mm; *P* = 0.0031), and *D. farinae* (*n* = 46 positives, median diameter: 5 mm, IQR: 3–6.3 mm; *P* = 0.0076) were significantly larger than those induced by the cockroach extract (Figures 3C–E). Representative examples of HDM and cockroach skin reactivity are presented in Figure 4.

### 3.4 Comparative analysis of allergen recognition in cockroach- and HDM-sensitized patients

**Figure 5:**
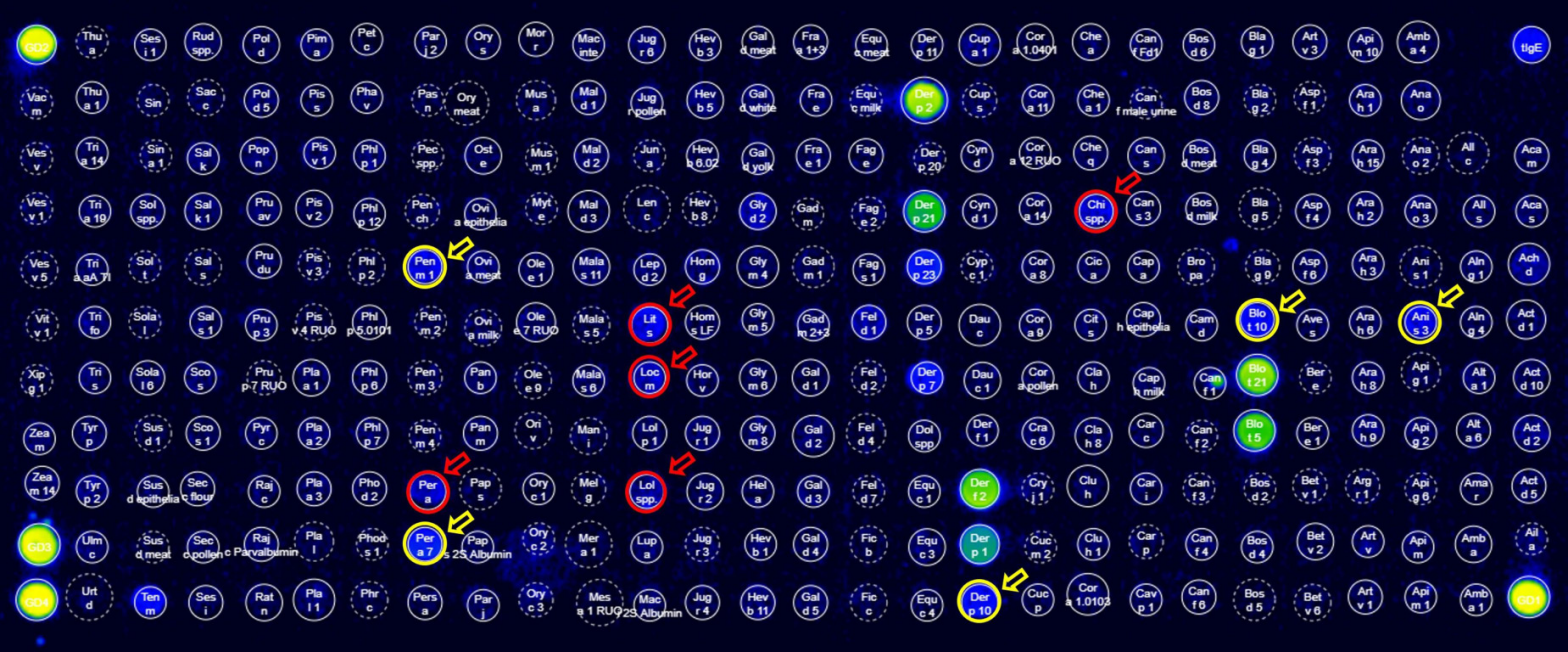
Allergy Explorer 2 (ALEX2) test result of an asthmatic patient with *P. americana* sensitization (cohort 2). The circles and arrows in red indicate a positive result for the extract of an invertebrate, including the extract or *P. americana*. The circles and arrows in yellow indicate a positive result for tropomyosins.

**Figure 6:**
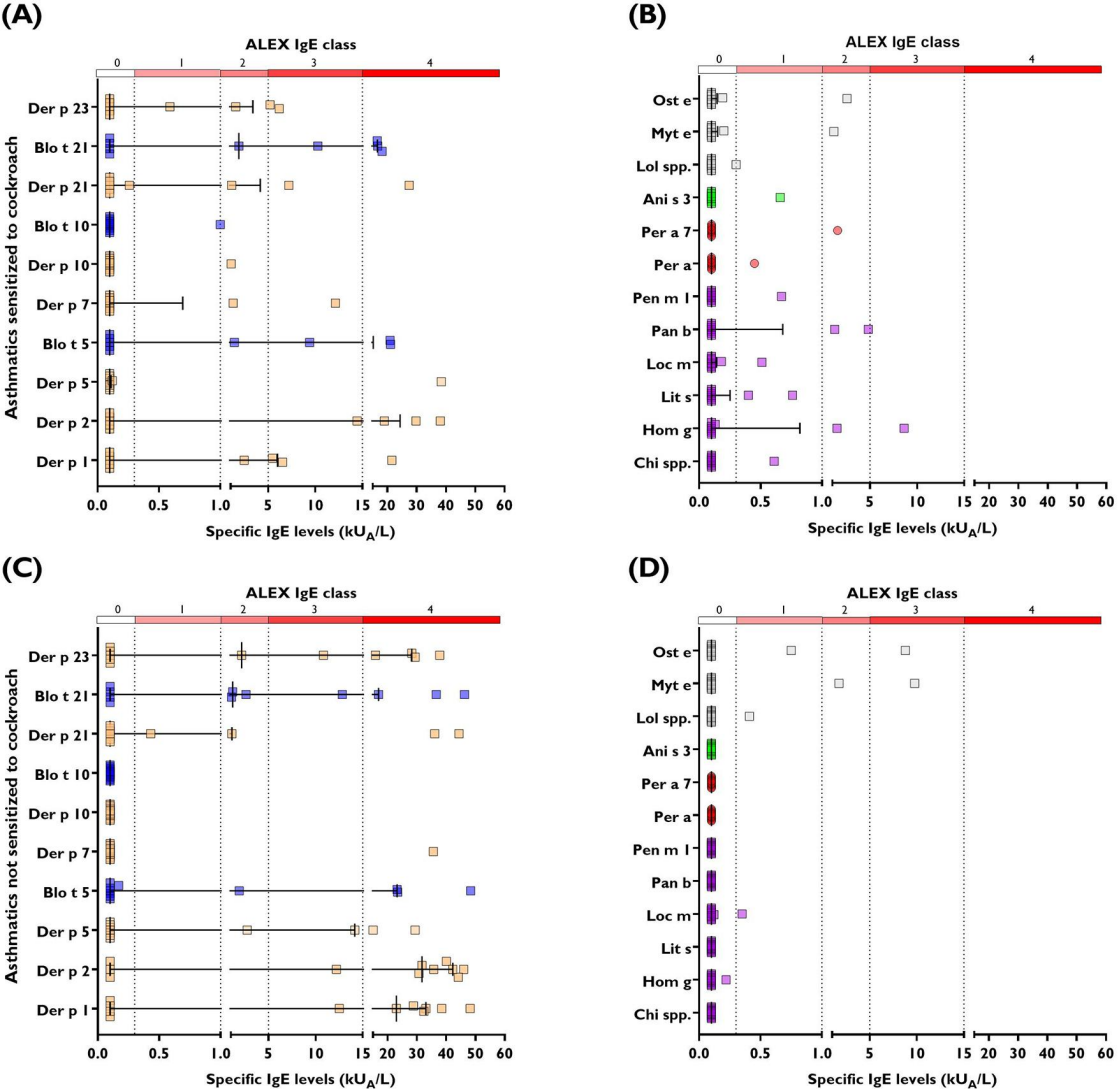
IgE classes according to plasma IgE levels determined by ALEX2 in nine asthmatic patients with a positive SPT result for *P. americana* (panel **A** and **B**) and 11 asthmatic patients with a positive SPT result for HDM but not sensitized to cockroach (panel **C** and **D**). The left panels indicate HDM components recognized by asthmatic patients and the right panels indicate IgE recognition to invertebrate allergens (all from cohort 2). Ani s 3, *Anisakis simplex*; Chi spp., *Chionoecetes* spp.; Hom g, *Homarus gammarus*; Lit s, *Litopenaeus* s.; Loc m, *Locusta migratoria*; Lol spp., *Loligo* spp.; Myt e, *Mytilus edulis*; Ost e, *Ostrea edulis*; Pan b, *Pandalus borealis*; Pen m 1, *Penaeus monodon*; Per a, *Periplaneta americana*; Per a 7, *Periplaneta americana* tropomyosin.

We next compared nine patients with a positive SPT result for cockroach and 11 with a positive SPT result for HDM (but with a negative result for cockroach) by using Allergy Explorer 2. We found that out of the nine patients with a positive SPT result for cockroach, one tested positive to cockroach allergens in Allergy Explorer 2 (Figure 5). This patient had a positive result for the cockroach extract (Per a) and for cockroach tropomyosin (Per a 7). Likewise, he tested positive for other invertebrate tropomyosins, including *Anisakis simplex* (Ani s 3), HDM (Blo t 10, Der p 10), and shrimp (Pen m 1). Even though this patient tolerated ingestion of shellfish, he tested positive for the extracts of blue shrimp (0.76 kU/L, *Litopenaeus* spp.), crab (0.61 kU/L, *Chionoecetes* spp.), and squid (0.30 kU/L, *Loligo* spp.) (Figure 5); he also had a positive test result for insects such as yellow mealworm (1.03 kU/L, *Tenebrio molitor*) and locust (0.51 kU/L, *Locusta migratoria*). All the other patients with a positive SPT result for cockroach tested negative for the cockroach extract (Per a) in ALEX2 but recognized allergens in HDM and other invertebrates (Figures 6A,B). The patients with a positive SPT result for HDM but with a negative result for cockroach showed an intense response to HDM components (Figure 6C) but less reactions to other invertebrates (Figure 6D). Overall, this confirmed a stronger response to HDM allergen components compared with cockroach and suggested that cockroach-sensitized patients may recognize other allergens in shellfish.

### 3.5 Evaluation of the clinical relevance of cockroach sensitization

To assess the clinical relevance of cockroach sensitization, demographic and clinical variables were compared between asthmatic patients with cockroach sensitization (*n* = 65) and those sensitized to HDM but not to cockroach (*n* = 120) (see Table 1). We found no significant differences in age, sex, BMI, FeNO, eosinophil counts, or socioeconomic stratum between cockroach-sensitized and non-sensitized patients. With regard to lung function parameters, no differences were found in FEV_1_, FVC, or the FEV_1_/FVC ratio between cockroach-sensitized and non-sensitized patients. In both groups, ACT and ACQ-5 scores reflected a good asthma control and no significant differences were found. Finally, 23.1% of patients reported symptoms of shellfish allergy among cockroach-sensitized patients compared with 11.7% in non-sensitized patients.

**Table 1:** Demographic and clinical characteristics of asthmatic patients sensitized to cockroach and HDM.

Variables	Sensitized to *P. americana* (*n* = 65)	Sensitized to HDM (*n* = 120)	*P*-value
Age, mean ± SD	39 ± 14	36 ± 13	0.17
Gender, female [*n* (%)]	49 (75.3)	93 (77.5)	0.8
Low socioeconomic status [*n* (%)]	54 (83.1)	97 (80.8)	0.8
Access to sewer service [*n* (%)]	59 (90)	109 (91)	0.9
Access to garbage collection [*n* (%)]	64 (98)	115 (96)	0.6
FeNO (ppb), median (IQR)	42 (18–72)	46 (29–79)	0.14
Blood eosinophils (cells/mm^3^)	220 (140–340)	290 (150–485)	0.09
FEV_1_ (% predicted), median IQR	79 (70–91)	79 (66–89)	0.5
FVC (% predicted), median IQR	91 (83–102)	88 (78–97)	0.2
FEV1/FVC, median IQR	0.74 (0.65–0.80)	0.75 (0.68–0.83)	0.4
ACT, mean ± SD	21 ± 3.7	20 ± 4.2	0.2
ACQ-5, mean ± SD	0.58 ± 0.72	0.94 ± 1.3	0.06
Self-reported allergy to shellfish (%)	15 (23.1)	14 (11.7)	0.06^a^

a Fisher’s exact test.

**Figure 7:**
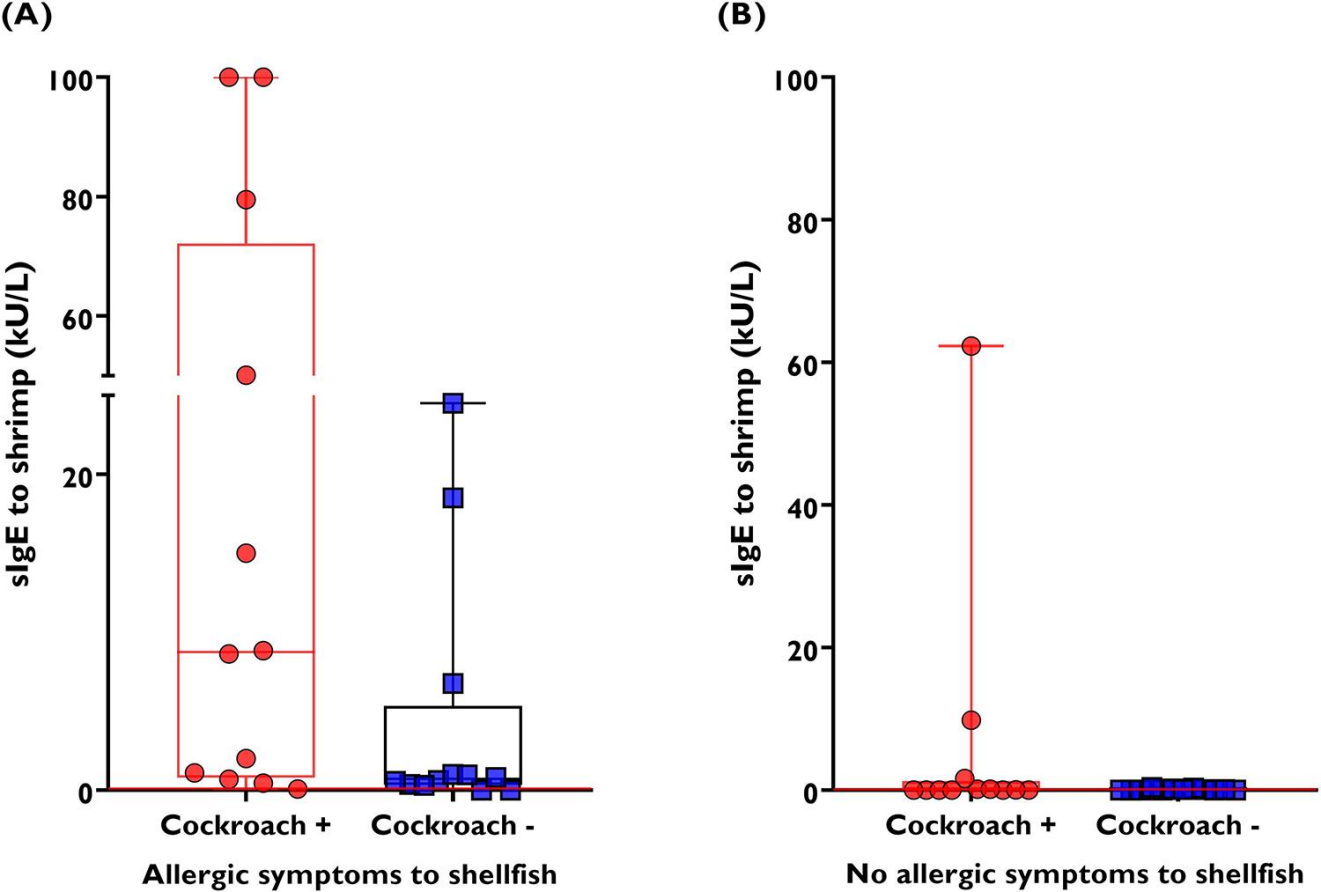
Specific IgE levels to shrimp in asthmatic patients (cohort 2) according to the presence of cockroach sensitization. **(A)** sIgE levels to shrimp determined by ImmunoCAP™ in patients with a previous history of shellfish allergy. **(B)** sIgE levels to shrimp determined by ImmunoCAP™ in patients without a history of shellfish allergy.

A comparative analysis of 24 samples from patients with shellfish allergy and 24 non-allergic to shellfish showed that IgE levels to shrimp were significantly higher in patients who were positive for cockroach (8.7 kU/L IQR 0.79–72 kU/L) compared with those who were negative for cockroach (0.71 kU/L, IQR 0.32–5.3 kU/L, Mann–Whitney test, *P* = 0.037) (Figure 7A). In patients without shellfish allergy, shrimp IgE levels were low and did not differ according to cockroach sensitization (Figure 7B). Among patients with shellfish allergy, we also found a significant and direct correlation between cockroach wheal diameter and specific IgE levels to shrimp (rho = 0.42, *P* = 0.003, effect size Fisher *z* = 0.45). There was no correlation between wheal diameters to HDM and IgE levels to shrimp (rho = 0.26, *P* = 0.22). Linear regression adjusted by age and gender confirmed a significant association between wheal diameter to cockroach and shrimp-specific IgE levels (coefficient 0.53, *t* = 4.28, *P* < 0.001).

## 4 Discussion

In this study, we demonstrate that HDM allergens induce a stronger IgE reactivity than cockroaches. This could be explained by an adjuvanticity in the HDM allergens or in the dosage and duration of exposure to HDM in bedrooms, because Cartagena is a tropical city with hot and humid conditions throughout the year. Even though cockroach allergens may not be the most relevant sensitizers, our study found that in patients with shellfish allergy, those with a positive SPT result for cockroach had higher IgE levels to shrimp (Figure 7). Also, in our sample, cockroach-sensitized patients recognized a wider repertoire of invertebrate allergens compared with HDM-sensitized patients without cockroach sensitization (Figure 6). Our observations replicate previous epidemiological data suggesting that cockroach sensitization may be more related to shrimp sensitization than to HDM (43) and confirm the direct and significant correlation between specific IgE levels to shrimp and cockroach (44).

Shrimp, cockroaches, and HDM are all arthropods, but their genetic distance varies, with shrimp and cockroaches being closer to each other (both in phylum Arthropoda, class Insecta, and Crustacea) than to mites (which are arachnids) (6, 45). Several studies have confirmed the cross-reactivity among these arthropods (8, 45, 46) and specific IgE against tropomyosin accounts for approximately 24% to 55% of the variability observed in specific IgE to clam, crab, German cockroach or shrimp (8). Previous inhibition studies have demonstrated IgE cross-reactivity between shrimp and cockroach. For instance, Atlantic shrimp extract (*Pandalus borealis*) inhibited IgE binding to German cockroach extract and vice versa. Also, by blot inhibition, the IgE binding capacity to cockroach was completely abolished by shrimp extract, while the IgE binding capacity to shrimp was partially inhibited by incubation with cockroach extract (47). Yang et al. evaluated IgE cross-reactivity between extracts from shrimp, HDM, and cockroach in 23 Guangzhou and 20 Shaoguan shrimp-sensitized subjects and reported that cockroach extract highly inhibited IgE binding to HDM and shrimp in subjects of rural areas, with the inhibition rates >85% (48). In that study, HDM extracts almost completely inhibited shrimp-specific IgE in urban subjects, but shrimp extracts could not fully inhibit dust mite–specific IgE, suggesting that dust mite might be a primary sensitizer in urban areas. However, cockroach allergen extracts had a significantly higher inhibition rate on the binding of IgE to HDM allergens in sera from rural subjects compared with urban subjects (48). Other studies have also confirmed allergenic cross-reactivity between HDM, cockroach, and shrimp allergens, but the level of cross-reactivity can vary depending on the population (3). *P. americana* tropomyosin (Per a 7) shares 82% sequence identity with shrimp tropomyosin (Pen a 1), and patient sera recognizing cockroach tropomyosin also bind to shrimp tropomyosin, implying strong structural and immunological homology (31). This molecular cross-reactivity was later confirmed by several other studies (46, 49, 50).

An interesting finding of this work was that cockroach-sensitized patients recognized the extracts of crustaceans, mollusks, and other shellfish allergens in the ALEX2 array, even though most patients were tolerating shellfish (Figure 6). Previous studies have proposed that tropomyosin acts as a cross-sensitizing pan-allergen among invertebrate species such as crustaceans, cockroaches, and HDM (9, 46). However, in our sample, only one patient recognized cockroach tropomyosin (Per a 7) in the ALEX2 array, and any of the patients tested showed a positive result for arginine kinases (Der p 20 or Bla g 9), which are considered pan-insect/crustacean cross-reactive allergens (42, 51). Overall, this suggested that other invertebrate allergens might be involved in this cockroach/shellfish cross-reactivity (48), for instance, myosin light chain (Bla g 8), troponin C (Bla g 6), hemocyanin (Bla g 3), or enolase, previously described as components of potential cross-reactivity between cockroach and shrimp (52). Interestingly, sera samples analyzed in this study reacted to crustacean extracts but did not recognize the homologous aforementioned allergens in Pen m 2 (arginine kinase), Pen m 3 (myosin light chain), and Pen m 4 (sarcoplasmic calcium-binding protein) placed in the array. In addition, IgE cross-reactivity between *B. germanica* and *P. americana* has been described (30), but here, we did not detect IgE antibodies to the components of *B. germanica* (Bla g 1, Bla g 2, Bla g 4, Bla g 5, and Bla g 9) in sera from any of the patients who tested positive for *P. americana*. Because the ALEX2 array does not contain Blo t 8, Der p 8, or Per a 5, it was not possible to evaluate a possible cross-sensitization with cockroach glutathione transferases (17).

Cockroach extracts show a high degree of molecular heterogenicity, so we cannot rule out that our patients may be recognizing other cockroach components (53), yet unidentified or still not present in this component-resolved diagnosis platform (8). Further studies are needed to elucidate which allergens are being recognized in crustacean's extracts by sera of cockroach-positive patients. Also, a careful interpretation must be done when counseling patients about cross-reactions to shellfish, especially when IgE sensitization is detected but is not clinically relevant.

It should be pointed out that method sensitivities may have influenced the results of this study since 41 patients recognized the American cockroach extract in ImmunoCAP at a cutoff level of ≥0.1 kU/L, and 24 remained still positive at a cutoff of ≥0.35 kU/L, decreasing the frequency of sensitization from 40.2% to 23.5%. Considering that ALEX2 implements an immunoenzyme method to detect IgE and the results are semiquantitative, its sensitivity and cutoff of ≥0.30 is not comparable to that of ImmunoCAP, and it is possible that low IgE levels to cockroach (0.10–0.35 kU/L), although positive, precluded the detection of cockroach molecular components in ALEX2. On the other hand, potential differences in linear and conformational epitopes between different platforms may have influenced our results, as previously described for food allergens (54).

IgE sensitization to *P. americana* extract in allergic individuals was first detected in New York (USA) (55), and cockroach allergens have been recognized as a significant trigger of asthma and allergic rhinitis in temperate regions (20). Our results are similar to those reported by Sun et al. in 6,304 patients with bronchial asthma and/or rhinitis, aged 5–65 years, who participated in the CARRAD study (56). It was found that 25.7% of the patients were SPT positive to the American cockroach, and that 88% of cockroach-sensitized patients were also positive to *D. pteronyssinus*. Cross-inhibition studies showed that only two out of 18 patients (11%) had cockroach as the primary sensitizing agent and that up to 50% of specific IgE detection could be due to cross-reactivity (56). A more recent study also reported a high cross-reactivity between cockroach and HDM allergens (3) and demonstrated that patients sensitized to *P. americana* showed a high cosensitization with HDM (*B. tropicalis* 90.7%, *D. pteronyssinus* 76.7%, and *D. farinae* 60.5%). Studies in the temperate regions of Central Europe with low HDM exposure have shown true IgE reactivity to cockroaches' molecular components (Bla g 1, Bla g 2, and Bla g 5) in only 0.6% of cases (8/1,255 patients), and nearly all of them were cosensitized with other non-cockroach components, including HDM allergens (in four patients). IgE reactions to inhaled tropomyosins were not that common (mite and cockroach derived) and represented only 1.9% for Der p 10 (28/1,255 patients) and 1.5% for Bla g 7 (19/1,255 patients) (57). In our study, with a high percentage of HDM sensitization, we also observed scarce IgE binding to the dominant cockroach allergens and low IgE levels to cockroach. Our patients usually report seeing cockroaches in their houses, but the use of carpets, rugs, and wallpaper is very rare, and doors and windows are regularly open during the day; therefore, particular aspects in the living conditions may have influenced the dosage and duration of cockroach exposure.

Previous studies have also shown that cockroach allergy is associated with exacerbations and asthma symptoms (26, 58). However, we found no difference in lung function measurements, the levels of type 2 inflammation biomarkers, or in the scores of asthma control between cockroach-sensitized and non-sensitized patients (Table 1), suggesting that in this cohort, cockroach sensitization was not associated with severe clinical outcomes. In addition, although this cohort had a marked African descent, and most participants were of low-income households, we could not replicate that cockroach sensitization could aggravate asthma. Based on wheal sizes and specific IgE levels measured in this study, we confirmed that HDM allergens were the primary sensitizers in this population, and IgE sensitization to cockroach may be a result of cross-reactivity. Considering the low IgE reactivity to cockroach extracts, the sensitization detected in our study may be considered a secondary sensitization. Among the limitations of this study, we must acknowledge a small sample size analyzed by ALEX2, possible misclassification from low-level IgE readings, and the cross-sectional design that precludes causal inference.

In conclusion, this study showed that IgE response to HDM was stronger than that to cockroach. There were no clinical differences between cockroach-sensitized and non-sensitized patients, and in this population, we did not observe an association between IgE sensitization to cockroaches and worsened asthma control or lung function. We found that skin wheal size to cockroach extract correlated with higher shrimp-specific IgE in patients reporting shellfish allergy. In clinical practice, cockroach sensitization co-occurs with HDM sensitization and also with a low level of IgE sensitization to other invertebrates, potentially serving as an indicator of those patients with pan-invertebrate cross-reactivity. However, where component-resolved data suggest cross-reactivity, clinicians should be cautious in interpretation, since molecular/serological evidence of shared epitopes is suggestive but does not confirm clinical cross-reactivity without a challenge test or clinical correlation. Therefore, care must be exercised when counseling patients since these sensitizations may not have clinical relevance. Although less common, monosensitized patients to cockroaches can be found in tropical settings, and the clinical relevance of this sensitization should be confirmed by nasal or conjunctival provocation, together with the evaluation of cockroach IgE sensitization *in vitro* using the extract and immunodominant components with highly sensitive methods. Future inhibition studies are needed to further elucidate HDM–cockroach cross-reactivity vs. cosensitization.

## Data Availability

The raw data supporting the conclusions of this article will be made available by the authors without undue reservation.
